# Flow cytometric approach to evaluate the impact of hydro-technical concrete compounds’ release to the freshwater microbiome

**DOI:** 10.1007/s10661-021-09481-5

**Published:** 2021-10-07

**Authors:** Barbara Wojtasik, Małgorzata Zbawicka, Lucyna Grabarczyk, Wojciech Juzwa

**Affiliations:** 1 Department of Genetics and Biosystematics, Faculty of Biology, University of Gdańsk, ul. Wita Stwosza 59, 80-308 Gdańsk, Poland; 2 Department of Genetics and Marine Biotechnology, Institute of Oceanology, Polish Academy of Sciences, ul. Powstańców Warszawy 55, 81-712 Sopot, Poland; 3 Department of Mechanics of Materials and Structures, Gdańsk University of Technology, ul. Gabriela Narutowicza 11/12, 80-233 Gdańsk, Poland; 4 Department of Biotechnology and Food Microbiology, Poznan University of Life Sciences, ul. Wojska Polskiego 28, 60-627 Poznan, Poland

**Keywords:** Microbial corrosion, Cellular physiology, Fluorescent staining, Bioadhesion

## Abstract

The aim of this research was to test the potential of applying a flow cytometric procedure to evaluate the impact of concrete compounds’ release to the freshwater microbiome. Cells from the collected samples were stained with a fluorogenic redox indicator dye that measures the redox potential of microbial cells. This novel approach was combined with the assessment of microorganisms’ penetration into the internal structures of concrete using the Rose Bengal sodium salt staining. Rose Bengal staining revealed an intense fouling of the upper and side walls of the concrete cubes and also indicated the penetration of microorganisms inside the concrete as observed for the cubes’ cross-sections. Flow cytometric cellular redox potential measurement revealed high percentages of active cells within the concrete’s porous structures and in non-exposed water (32.7% and 30.2% of active cells) versus samples from exposed water and concrete’s outer surfaces (6.8%, 6.1%, and 3.3% of active cells). The results demonstrated a detrimental impact of hydro-technical concrete on the vitality of microbial cells within the freshwater environment. Tested protocol by analyzing the physiology of microbial cells improved the functional description of complex communities to evaluate the fate of contaminants present in the concrete-based hydro-technical infrastructure.

## Introduction

Traditional concrete components such as cement and mineral aggregate do not pose a threat to the environment. Recently, increasing changes in concrete technology and the use of new components are being observed. Specific chemical additives and admixtures adequate to outside conditions and the size of the concrete elements are used (Horszczaruk & Brzozowski, 2014). Normal concrete consists of cement, mineral aggregate, and water as well as possible liquefaction admixtures. It is laid in air conditions, in a form or boarding (Neville, 2011).

Hydro-technical concretes usually contains mineral additives (e.g., fly ash), anti-washout admixtures (AWA), and superplasticizers, e.g., high-range water-reducing admixture (HRWR), increasing the washout resistance of underwater concretes, keeping the water in the concrete mixture to prevent high leaching of cement paste (Heniegal et al., 2015), and providing with high durability (Khayat & Sonebi, 2001). The modified composition of the concretes can be dangerous to living water organisms, e.g., Bivalvia (Wojtasik et al., 2019). The bactericidal effect of some additives like silica-titania—special nanocomposite—and also a positive effect of a waste glass aggregate for bactericidal properties of cement mortars were observed (Chung et al., 2017; Sikora et al., 2017; Wei et al., 2013). Concrete corrosion can be caused by chemical and physicochemical, thermal, mechanical, and biological. Biological corrosion concerns the development of algae, fungi, and bacterial strains on the concrete surface. In itself, the occurrence of these organisms is not something extremely dangerous. Unfortunately, the additional presence of some sulfur, nitrogen, or carbon compounds can cause bacteria to start producing various types of harmful acids or other substances that promote chemical corrosion (Valix et al., 2012). Perez et al. investigated the role of planktonic versus biofilm forms of microorganisms dwelling in the concrete structure. Their results demonstrated that the microbial cells forming a biofilm structure on the cementitious material may result in the volatile fatty acids–induced biodeterioration of concrete (Perez et al., 2021).

The chemical admixtures of modern hydro-technical concretes are potentially dangerous, especially in concrete mixtures laid underwater. This promotes the migration of chemical compounds into the environment. The modified composition of concrete, even after setting, can be dangerous to living organisms when in constant contact with water, often rippling (Khayat & Sonebi, 2001). Thus, the concrete constructions and the addition of different new components to the concrete have an often unknown impact on the aquatic environment. There is very little research related to this issue, e.g., Wojtasik et al. who have described the harmful effects of hydro-technical concrete on freshwater Bivalvia (Wojtasik et al., 2019).

Although the microbial-enhanced concrete corrosion is commonly considered a serious problem concerning hydro-technical infrastructure, recently, a part of the attention is directed towards the deleterious impact of concrete compounds on microorganisms dwelling within freshwater environments. Microbes colonizing different environmental niches exhibit substantial structural and functional variability resulting from complex interactions driven by environmental changes sensed by individual cells residing in distinct locales (Davis & Isberg, 2016). The heterogeneity of environment-associated microbes is reflected by the different physiological states of microbial cells. It should be emphasized that the ability to distinguish between live, dead, dormant, and sub-lethally injured cells enables to gain a more comprehensive view of cellular activity. The selection of a technique that is appropriate for the evaluation of the condition of microbial cells exposed to the specific components of hydro-technical concretes is a challenge. Flow cytometry-based approaches represent promising experimental protocols providing an accurate insight into the diversity of complex microbial populations (Díaz et al., 2010). Flow cytometric measurement (FCM) enables to discriminate in situ the different physiological states of microorganisms at the single-cell level (Joux & Lebaron, 2000; Nebe-Von-Caron et al., 2000).

This study aimed at developing a novel method with potential for use in environmental biology research to determine the fate of contaminants present in the concrete-based hydro-technical infrastructure. The approach we developed enabled us to collate the free-living microbes with the adherent ones, associated with the surface of hydro-technical concrete. The samples thus comprised both, planktonic and associated with the concrete surface microbial cells. The significant advantage of this study was the prospect of analyzing the physiology of planktonic and concrete-associated microbes. The main objective was to investigate the impact of the concrete components on the vitality of microbial cells within a freshwater microbiome exposed to the compounds of hydro-technical concrete.

## Materials and methods

### Experimental design

#### Components

The concrete samples, marked as BP, were prepared using natural pebble aggregate, portland cement, and fugacious siliceous ash (both used as a binder). The components used to prepare concrete samples and the chemical composition of concrete components are listed in Tables 1 and 2. The hydro-technical concrete used for the experiment was prepared based on the generally available and universal procedure for hydro-technical concretes in laboratory conditions. The ingredients were combined in a forced mixing mixer. The samples were prepared in standard 100 × 100 [mm] cubic forms (cubes), ripened in conditions of increased humidity (approx. 98%), at a temperature of 20 °C. Mineral additive and AWA admixture were added to the concrete composition. This composition is typical for concrete laid underwater.

**Table 1:** The components used in concretes in the experiment

Concrete signature	BP component quantity [kg/m^3^]
CEM I 42.5R	320
Fugacious siliceous ash	80
Natural rinsed sand 0–2 mm	660
Natural rinsed gravel 2–16 mm	1140
Superplastificator FM	2.72
Anti-washout admixture	3.0
Water	175

**Table 2:** Chemical composition of concrete components (component share, weight [%])

	SiO_2_	Al_2_O_3_	Fe_2_O_3_	CaO	MgO	SO_3_	K_2_O	Na_2_O	TiO_2_	Loss of roast
Cement	19.6	5.1	3.1	63.0	1.0	2.9	-	-	-	2.6
Fugacious ash	46.9	26.6	7.2	3.1	2.1	0.9	1.2	1.0	-	-
Natural aggregate	89.9	3.8	1.8	0.6	0.35	0.02	1.4	0.5	-	1.4
AWA additive	1.73	0.17		3.83	0.07	0.5		0.57	-	50.28

#### Experiment, natural environment, and aquaculture

In order to reduce the toxicity of concrete, the cubes were placed in the lake (natural reservoir, about 1500 ha), in the coastal and wave zone, 5 m from the shore, at a depth of 1 m, for 9 months (from September 2016 to June 2017) (Mariak et al., 2018; Wojtasik et al., 2019). After the exposition period, BP cubes were stained with the Rose Bengal sodium salt (Sigma-Aldrich Merck) to determine the spreading of samples by small aquatic organisms and microorganisms. The concrete blocks were cut to assess the penetration of microorganisms into the concrete structure—the biological corrosion of the concrete. BP cubes after exposition were transferred to the aquariums (20-L tanks) containing water from the same lake with lake sediment in the base (3–4 cm) for the next 8 months. The aquariums were constantly aerated.

### Samples collection from aquaculture for microbiological analysis

Water samples were collected using a pipette (sample volume 2 mL), and swab samples (swabs of an area of 100 cm^2^ taken using sterile cotton) were placed in tubes with 1 ml of 1% PBS (phosphate-buffered saline) solution. Both types of samples were transferred to the Department of Biotechnology and Food Microbiology laboratory for direct staining and analysis.

Sample no. 1—water from aquaculture with concrete cube

Sample no. 2—sample containing swabs collected on the side wall

Sample no. 3—sample containing swabs collected on the underside wall

Sample no. 4—sample containing swabs collected from porous structures on concrete cube walls

Sample no. 5—water from control aquaculture, without the concrete cube

### Flow cytometric evaluation of microbial cells’ metabolic activity

To investigate the metabolic activity of microbial cells, a fluorescent staining using BacLight™ Redox Sensor™ Green Vitality Kit (Thermo Scientific, USA) was employed according to a method described previously (Duber et al., 2020). The kit consists of two components: propidium iodide (PI)—live/dead discrimination (cellular integrity)—and RedoxSensor™ Green reagent—redox potential-sensitive reagent (RSG) cellular metabolic activity measurement. RSG is a fluorogenic redox indicator dye which subjected to the conversion by microbial reductases (involved in electron transport systems within cells) and enables to measure the cellular redox potential (CRP) of microbes (Gray et al., 2005). Following excitation by a laser beam (488 nm), the converted dye emits green fluorescence, whose intensity is directly proportional to CRP levels, reflecting the metabolic activity (vitality) of microbial cells (Díaz et al., 2010).

Samples prior to staining and analysis were filtered using a nylon net 60-µm syringe filter (assembled with Swinnex filter holder 25 mm—both from Merck Millipore, Germany). The BD FACS Aria™ III (Becton Dickinson, USA) flow cytometer (cell sorter) was employed to analyze the vitality of microbial cells within the samples. The distinct and demonstrating low CV (coefficient of variation) values in measured parameter (CRP) sub-populations of microbial cells were defined by gating in the dot plots of green fluorescence (FITC detector from RSG) versus side scatter signals (SSC). Each sample was analyzed in triplicates. The calculation of CRP was performed using the medians of green fluorescence (FITC-A) signals. P4 (active), P5 (mid-active), and P6 (non-active) sub-populations of analyzed cells were defined based on the differences in the levels of metabolic activity measured as CRP. To each sub-population, the arbitrary selected mean CRP ranges were attributed as follows: 280–1000 relative fluorescence units (RFU), 1500–7500 RFU, and 8700–70,000 RFU for P4 (non-active), P5 (mid-active), and P6 (active) sub-populations, respectively.

## Results and discussion

**Fig. 1:**
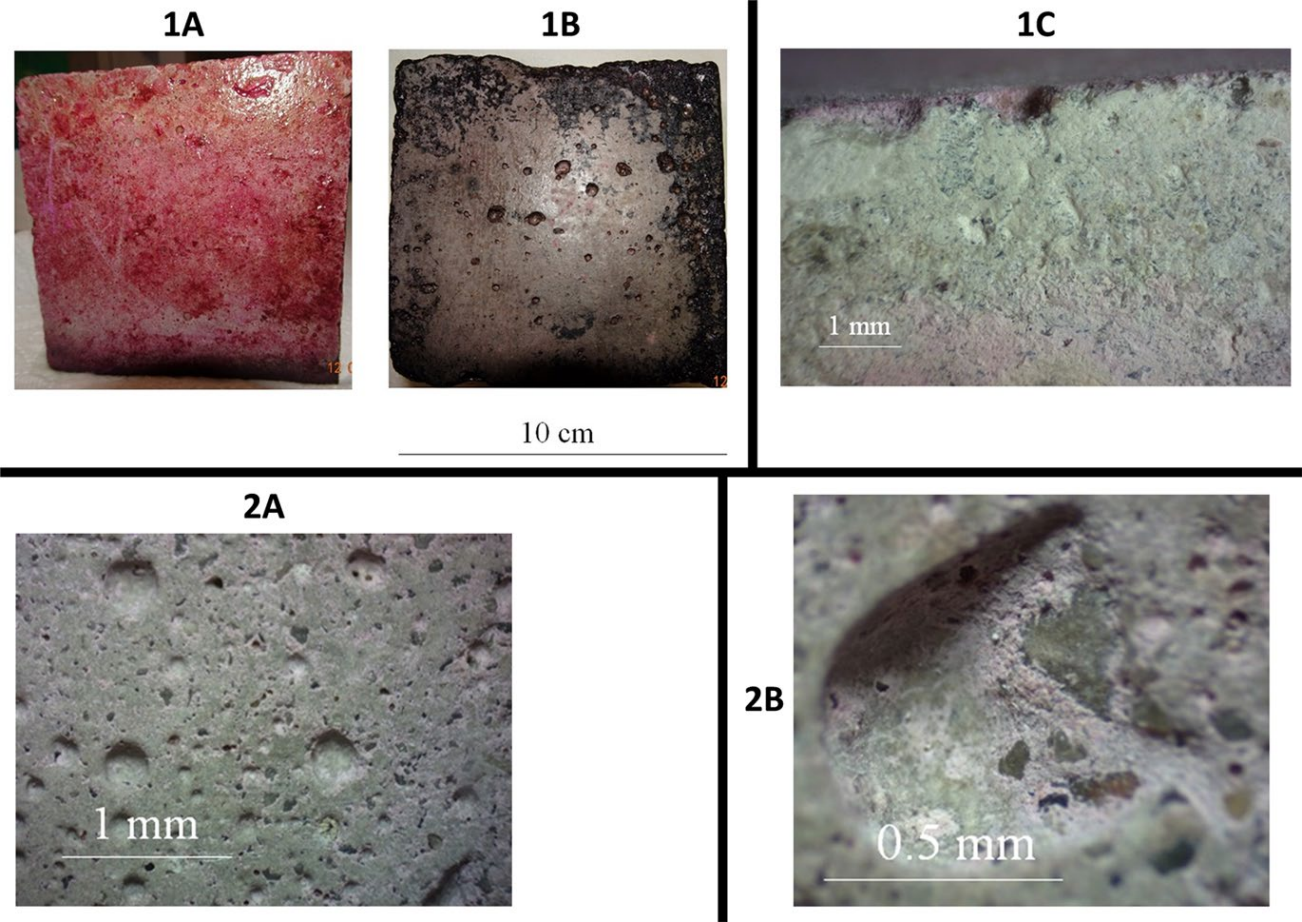
Image of concrete BP cubes after incubation in a lake for 9 months (Rose Bengal–stained surface of external BP). Side wall **1A**. Underside **1B**. Microscopic images of BP concrete surfaces after incubation in the natural environment (lake) for 9 months, Bengal Rose staining and concrete drying: wall cross-section (magnification × 10) **1A**, the upper surface of the wall (magnification × 7) **1B**, and the upper surface of the wall (magnification × 20) **1C**

After 9 months of BP cube exposition to lake water, the Rose Bengal staining was performed to reveal the concrete interaction and observed that concrete cubes strongly interacted with BP-associated microbes. The penetration of microorganisms into the internal structures of concrete varied, and deposition outside was evaluated (Fig. 1 (1A)). Intense fouling of the upper and side walls of the cubes was observed. The underside surface was less overgrown (Fig. 1 (1B)). Cutting the cube revealed the penetration of microorganisms inside the concrete (Fig. 1 (1C)). The upper surface of the wall image after Bengal Rose staining and concrete drying enlarged 7 and 20 times and is presented in Fig. 1 (2A and 2B).

**Fig. 2:**
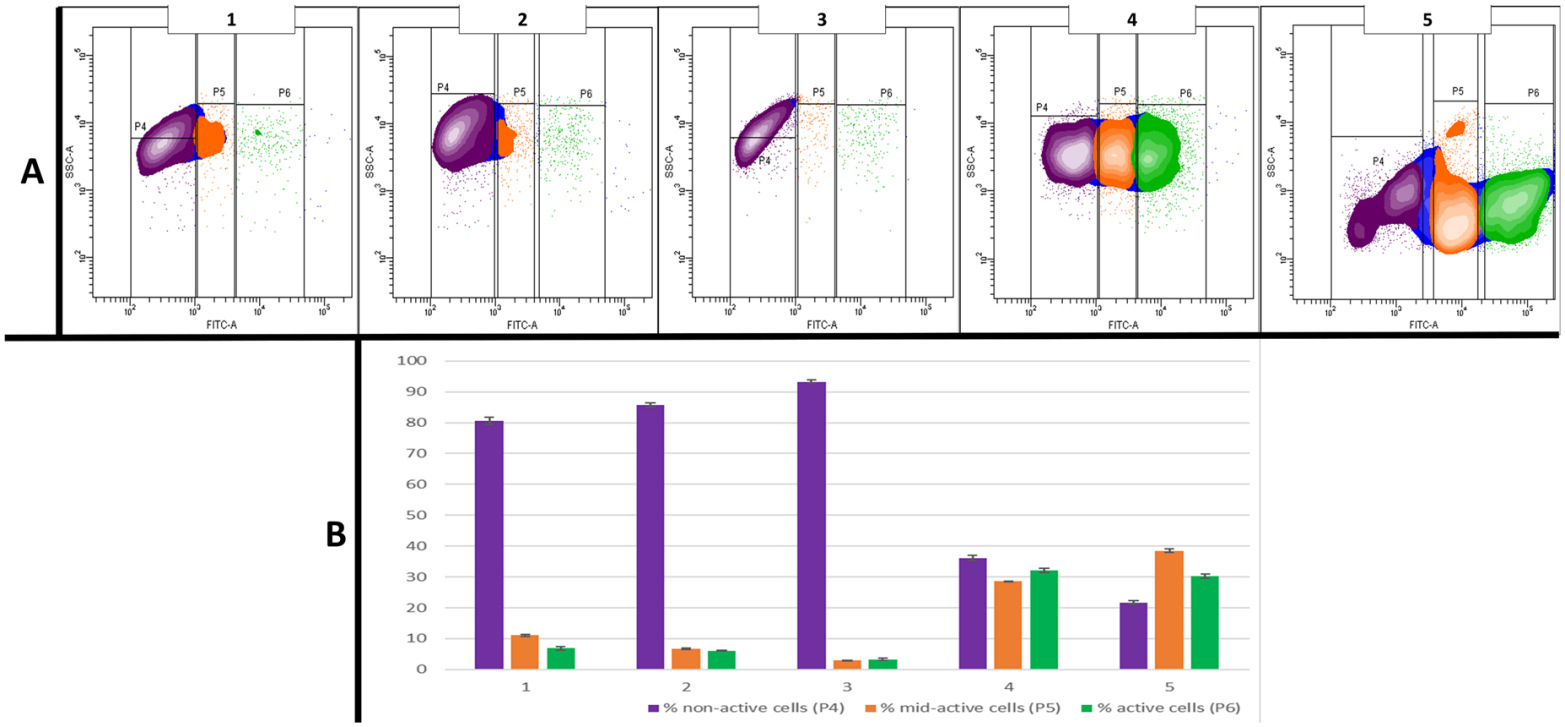
Flow cytometric analysis of the metabolic activity of microbial cells from concrete’s cube exposed and non-exposed samples, designated as follows: sample 1—water from aquaculture with a concrete cube; sample 2—sample containing swabs collected on the side wall; sample 3—sample containing swabs collected on the underside wall; sample 4—sample containing swabs collected from porous structures on concrete cube walls; and sample 5—water from control aquaculture without the concrete cube. The active, mid-active, and non-active microbial sub-populations (P4, P5, and P6, respectively) were discriminated. The analysis demonstrated the differences in the percentages of microbial cells from each sub-population in relation to the concrete exposition and the sample origin. The sub-populations P4, P5, and P6 were defined on bivariate dot plots displaying fluorescence intensity signals from FITC (*x*-axis) vs. SSC (*y*-axis) detectors, corresponding to cellular redox potential vs. side scatter measurement (diagrams in **A**). The discrimination of each sub-population was based on the differences in cellular redox potential values (CRP). CRP values are the fluorescence signal intensities measured by the specific light detector and expressed as relative fluorescence units (RFU). Percentages of active, mid-active, and non-active cells are shown in diagram **B**

Concrete cubes after exposition were transferred to tanks of water from the lake (aquariums). During the experiment, the measurements of pH and electrolytic conductivity (C) of water in the tanks were performed. The temperature was about 20 °C. Basic physical–chemical parameters pH = 7.667 and C = 251 μS/cm indicated I class of according to the current standards (Water Directive—Dz.U. 257, poz. 1545, 09.11.2011). After exposition, a significant increase of electrolytic conductivity (from 251.0 to 1570 µS/cm) was observed, and pH value increased slightly, while the oxygen level dropped significantly (from 86.7 to 18.7% of O_2_).

Flow cytometric measurement (FCM) of fluorescently labeled microbial cells from tested samples provided a fingerprint of the cellular vitality, which expands the concept of live and dead cell discrimination. It enables the evaluation of the metabolic activity of microbial cells by the measurement of cellular redox potential (CRP). The CRP enabled the discrimination between different physiological states of microbial cells from tested samples to resolve the non-active (P4), intermediate (P5), and active (P6) bacterial sub-populations (Fig. 2). Thus, the heterogeneity within the complex microbial community has been revealed. The percentages of the defined sub-populations reflected the physiological heterogeneity of microbial cells within the tested samples and enable us to assess the influence of concrete’s compounds on microbial cells’ vitality as measured by cellular metabolic activity. As demonstrated in Fig. 2, water from aquaculture with concrete cube, side, and bottom cube walls, samples 1, 2, and 3 respectively, demonstrated the prevalence of non-active microbial cells (80.53, 85.73, and 93.23%, respectively). The reference sample, containing water from control aquaculture without the concrete cube (sample 5), showed the following percentages of non-active, mid-active, and active cells: 21.73, 38.57, and 30.2%, respectively. Surprisingly sample 4 (sample collected from porous structures on concrete cube walls) demonstrated similar to reference sample distribution of non-active, mid-active, and active microbial cells: 36.17, 28.67, and 32.07%, respectively.

As demonstrated in diagram B of Fig. 2, the results for samples 4 and 5 where high percentages of active and mid-active cells were accompanied by low levels of non-active cells correlated with the prevalence of non-active cells in samples 1, 2, and 3. The presence of high levels of active cells within the concrete’s porous structures (sample 4) and in non-exposed water (sample 5) and the simultaneous inhibition of cellular activity within samples from exposed water (sample 1) and concrete’ outer surfaces (sample 2 and 3) indicate the detrimental impact of concrete’s components on the vitality of microbial cells.

The staining of microbial cells with RSG revealed the analytic potential to evaluate the metabolic activity at the single-cell level. Kalyuzhnaya et al. demonstrated that the reagent does not affect the metabolism of bacterial cells and may serve as a reliable marker for studying the physiology of microbial cells (Kalyuzhnaya et al., 2008). The potential to resolve the non-active, mid-active, and active bacterial sub-populations based on the CRP measurement was verified using reference analysis involving 1:1 mixtures of heat-inactivated and non-treated bacterial log-phase cultures (Juzwa et al., 2016). The robustness of the CRP measurement was also demonstrated using the specific grow-back test. It involved a combination of single-cell sorting and colony counts demonstrating that CRP measurement correlates with the growth potential of the defined sub-populations of *Pseudomonas aeruginosa* cells (Juzwa et al., 2018).

Concrete constructions containing new components, improving the concrete robustness to the environmental conditions, are considered to have an often unknown impact on the aquatic environment. Studies on the influence of concrete’s components on the environmental microflora are limited in number and in terms of methods applied to measure the impact on the microbiome at a single-cell level. Mariak et al. presented the description of different kinds of hydro-technical concrete as a potential habitat of water organisms and that some underwater concretes (UWC) could be toxic for living organisms (Mariak et al., 2018). In contrast, the problem of microbial cell-induced concrete corrosion is commonly investigated (Grengg et al., 2018). Recent studies of Wojtasik et al., who observed the lethal effect of two types of freshly hardening concrete on commonly occurring freshwater hydrobiont, demonstrated the significant detrimental influence of underwater concrete components on freshwater Bivalvia (Wojtasik et al., 2019).

Recently, a number of studies have noted yet another aspect of microbial interaction with the environment. It was demonstrated that bacteria can also have a positive effect on the properties of concrete and in the repair of concrete cracks (Osman et al., 2021). These authors suggested a new technique enabling the self-healing of cracks in concrete by the microbiologically induced calcium carbonate precipitation (MICCP) in concrete by the bacteria *Bacillus subtilis*. It was found that the introduction of the bacterial cell to the mixing water of concrete has a positive effect on the strength properties of concrete. Microbial self-healing concrete has also been used in water conservation projects (Osman et al., 2021). Osman et al. (2021) investigated the curing concrete in Qaroun lake conditions. They found that the activity of bacteria (*Bacillus* sp.) and algae (*Dunaliella salina*) also contributed to the protection of the reinforcement layer and corrosion inhibition (Osman et al., 2021).

The protocol that we developed demonstrated the degree of colonization and deposition of bacteria on the outside, and penetration inside the concrete structure. FCM expanded the results of routine diagnostic methods and resolved the limitations of CFU counting. Being a culture-independent approach, it enabled the discrimination between active, non-active (including dead cells), and mid-active, including the viable but non-culturable cell (VBNC) forms of microbial cells (Juzwa et al., 2018). The application potential of the described method in environmental monitoring involves the evaluation of the corrosion kinetics in combination with measuring to what extent the spread of toxic compounds from hydro-technical concretes may affect the freshwater microflora. Staining with Rose Bengal sodium salt allows to determine the areas of occurrence of living microorganisms, and flow cytometric test allows to determine their physiological condition at the quantitative level. The novelty of this approach results from the involvement of the modern single cell–based instrumental technique enabling the functional characterization of complex microbial communities within environmental samples.

## Conclusion

In this study, a Rose Bengal sodium salt staining to assess the microorganisms’ penetration into the internal structures of concrete was combined with a single cell–based approach—fluorescent staining and FCM. Flow cytometry was applied to measure the cellular metabolic activity and investigate the in situ dynamics of the planktonic and concrete-associated microbial cells within the complex aquaculture microbiome. The evaluation of the physiology of microbial cells revealed the potential to develop a routine protocol for assessing the degree of environmental contamination with reference to microbiological balance within different ecological niches. The tested protocol enabled us to evaluate the impact on the condition of environmental microbiomes exerted by the specific components of hydro-technical infrastructure. As the work refers to the toxicity of hydro-technical infrastructure to freshwater microbiome, the application potential of the developed procedure is strongly related to environmental diagnostics. The combination of Rose Bengal sodium salt staining and a single cell-based flow cytometric approach may provide a tool for the precise and fast detection of interferences within freshwater microbiome and diagnose the processes taking place on the surface and inside of concrete. The method allows for the standardization of measurements. The procedure may provide a new quality in the current works in the field of concrete technology, concrete-environment interaction, and in the analysis of the microbiome/consortia of microorganisms emerging for specific types of concrete.

## Data Availability

Some or all data, models, or codes that support the findings of this study are available from the corresponding author upon reasonable request (raw images of concrete surfaces, raw flow cytometric data—fcs files).

## Code Availability

Available from the corresponding author upon a request.
